# Genome Wide Association (GWA) Study for Early Onset Extreme Obesity Supports the Role of Fat Mass and Obesity Associated Gene *(FTO)* Variants

**DOI:** 10.1371/journal.pone.0001361

**Published:** 2007-12-26

**Authors:** Anke Hinney, Thuy Trang Nguyen, André Scherag, Susann Friedel, Günter Brönner, Timo Dirk Müller, Harald Grallert, Thomas Illig, H.-Erich Wichmann, Winfried Rief, Helmut Schäfer, Johannes Hebebrand

**Affiliations:** 1 Department of Child and Adolescent Psychiatry, University of Duisburg-Essen, Essen, Germany; 2 Institute of Medical Biometry and Epidemiology, Philipps-University of Marburg, Bunsenstr, Marburg, Germany; 3 Institute of Medical Informatics, Biometry and Epidemiology, University of Duisburg-Essen, Essen, Germany; 4 Forschungszentrum für Umwelt und Gesundheit (GSF), Institute of Epidemiology, Munich-Neuherberg, Germany; 5 Department of Clinical Psychology and Psychotherapy, Philipps-University of Marburg, Marburg, Germany; Innsbruck Medical University, Austria

## Abstract

**Background:**

Obesity is a major health problem. Although heritability is substantial, genetic mechanisms predisposing to obesity are not very well understood. We have performed a genome wide association study (GWA) for early onset (extreme) obesity.

**Methodology/Principal Findings:**

a) GWA (Genome-Wide Human SNP Array 5.0 comprising 440,794 single nucleotide polymorphisms) for early onset extreme obesity based on 487 extremely obese young German individuals and 442 healthy lean German controls; b) confirmatory analyses on 644 independent families with at least one obese offspring and both parents. We aimed to identify and subsequently confirm the 15 SNPs (minor allele frequency ≥10%) with the lowest p-values of the GWA by four genetic models: additive, recessive, dominant and allelic. Six single nucleotide polymorphisms (SNPs) in *FTO* (fat mass and obesity associated gene) within one linkage disequilibrium (LD) block including the GWA SNP rendering the lowest p-value (rs1121980; log-additive model: nominal p = 1.13×10^−7^, corrected p = 0.0494; odds ratio (OR)_CT_ 1.67, 95% confidence interval (CI) 1.22–2.27; OR_TT_ 2.76, 95% CI 1.88–4.03) belonged to the 15 SNPs showing the strongest evidence for association with obesity. For confirmation we genotyped 11 of these in the 644 independent families (of the six *FTO* SNPs we chose only two representing the LD bock). For both *FTO* SNPs the initial association was confirmed (both Bonferroni corrected p<0.01). However, none of the nine non-*FTO* SNPs revealed significant transmission disequilibrium.

**Conclusions/Significance:**

Our GWA for extreme early onset obesity substantiates that variation in *FTO* strongly contributes to early onset obesity. This is a further proof of concept for GWA to detect genes relevant for highly complex phenotypes. We concurrently show that nine additional SNPs with initially low p-values in the GWA were not confirmed in our family study, thus suggesting that of the best 15 SNPs in the GWA only the *FTO* SNPs represent true positive findings.

## Introduction

The advent of genome wide association studies (GWAs) already has had a major impact on the identification of polygenes involved in human body weight regulation [1]. However, a GWA based on obese cases and lean controls has not yet been described. GWA have recently proven extremely powerful for the detection of genes/SNPs for different complex disorders [2], [3]. The progress has been particularly impressive for type 2 diabetes mellitus (T2DM) [4]–[7]. *FTO* was one of the genes picked up in GWA studies for T2DM [5], [6], adjustment for BMI revealed that this effect was solely based on this quantitative phenotype [5]. We performed a GWA (Genome-Wide Human SNP Array 5.0; Affymetrix) on patient samples stemming from both ends of the BMI distribution and subsequently aimed to confirm the 15 GWA SNPs with minor allele frequency (MAF) ≥10% rendering the lowest p-values determined upon analysis of four genetic models (additive, recessive, dominant and allelic) in an independent family-based study.

## Results

The GWA was analysed for the four genetic models additive, recessive, dominant and allelic. By sorting all analysed SNPs with a MAF ≥10% by minimal nominal p-values a list was derived for the best 15 SNPs (Table 1). Six SNPs (rs1121980, rs9939973, rs7193144, rs9940128, rs8050136, rs9939609, pair-wise r^2^ range 0.88–1) in *FTO* were among these 15 best SNPs of the scan (see Table 1); all six SNPs localize to the same linkage disequilibrium (LD) block. *FTO*-SNP rs1121980 rendered the lowest nominal p-value of 1.13×10^−7^; this SNP was the only SNP that survived correction for multiple testing (corrected p = 0.0494; Table 1). The log-additive OR for the rs1121980 risk T-allele was 1.66 (95% CI 1.37–2.01); the odds ratios for heterozygotes (CT) and homozygotes (TT) were estimated at 1.67 (95% CI 1.22–2.27) and 2.76 (95% CI 1.88–4.03), respectively. Frequencies of the T-allele in cases and controls were 0.53 and 0.41 (Table 1).

**Table 1:** Top 15 SNPs associated with early onset extreme obesity from the Genome-Wide Human SNP Array 5.0 (lowest nominal p-values across four genetic models) and their confirmation using family-based association studies for the risk allele derived from the GWA data

chromo-some	nearest gene or cDNA^1^	SNP	GWA 500K case-control approach							confirmation-family-based association study^2^	
			dbSNP alleles (bold: obesity risk related)	minor allele frequency cases/controls [%]	genotype distribution cases[%]/controls[%]			odds ratio (95% CI) under best genetic model for minor allele	p-value (x10^−6^) (corrected empirical p-value)	risk allele (more frequently transmitted)	p-value (one-sided) (Bonferroni corrected p-value)
16	*FTO*	rs1121980	C/**T**	53.0/40.7	104 [21.5]/153 [34.6]	247 [51.0]/218 [49.3]	133 [27.5]/71 [16.1]	1.660 (1.369;2.017) log-additive	0.113 (0.0494)	T	0.0001 (0.0011)
16	*FTO*	rs9939973	**A**/G	52.7/40.7	104 [21.5]/153 [34.6]	250 [51.7]/218 [49.3]	130 [26.9]/71 [16.1]	1.644 (1.355;1.999) log-additive	0.211 (0.0875)	A	0.0003 (0.003)
16	*FTO*	rs7193144	**C**/T	50.1/38.7	121 [25.0]/165 [37.4]	241 [49.8]/211 [47.8]	122 [25.2]/65 [14.7]	1.593 (1.318;1.925) allelic	0.765 (0.2802)	-4	-
16	*FTO*	rs9940128	**A**/G	52.1/40.7	106 [21.8]/153 [34.6]	254 [52.3]/218 [49.3]	126 [25.9]/71 [16.1]	1.606 (1.324;1.954) log-additive	0.772 (0.2831)	-4	-
20	*C20orf75*	rs6076920	C/**G**	14.9/8.3	347 [71.4]/375 [84.8]	133 [27.4]/61 [13.8]	6 [1.2]/6 [1.4]	2.242 (1.601;3.157) dominant	0.86 (0.3081)	C	0.850 (1)
20	*none*	SNP_A-1967967^5^	**A**/G	14.5/7.7	348 [71.5]/375 [84.8]	137 [28.1]/66 [14.9]	2 [0.4]/1 [0.2]	2.236 (1.596;3.148) dominant	0.94 (0.3321)	G^3^	0.9742 (1)
16	*FTO*	rs8050136	**A**/C	50.0/38.7	123 [25.3]/165 [37.3]	240 [49.4]/212 [48.0]	123 [25.3]/65 [14.7]	1.585 (1.312;1.915) allelic	0.976 (0.3433)	-4	-
14	*TSHR*	rs3783950	C/**G**	43.7/54.9	151 [31.5]/88 [20.2]	239 [49.8]/216 [49.7]	90 [18.8]/131 [30.1]	0.635 (0.526;0.767) allelic	1.38 (0.4541)	G	0.132 (1)
4	*BC041448*	rs2969001	C/**G**	38.1/27.6	194 [39.8]/240 [54.3]	215 [44.1]/160 [36.2]	78 [16.0]/42 [9.5]	1.614 (1.320;1.974) allelic	1.60 (0.5044)	G	0.108 (1)
16	*FTO*	rs9939609	A/**T**	49.7/38.7	123 [25.4]/164 [37.1]	241 [49.8]/214 [48.4]	120 [24.8]/64 [14.5]	1.565 (1.295;1.892) allelic	1.94 (0.5681)	-4	-
4	*none*	rs619819	C**/G**	30.4/39.4	248 [51.0]/157 [35.5]	181 [37.2]/222 [50.2]	57 [11.7]/63 [14.3]	0.529 (0.402;0.694) dominant	1.96 (0.5717)	C	0.566 (1)
4	*none*	rs2172478	**A**/G	30.6/21.2	234 [48.1]/281 [63.6]	207 [42.6]/135 [30.5]	45 [9.3]/26 [5.9]	1.880 (1.433;2.467) dominant	2.33 (0.6348)	A	0.292 (1)
20	*PCSK2*	rs16998603	**A**/G	14.4/7.9	346[71.8]/371 [84.7]	133 [27.6]/65 [14.8]	3 [0.6]/2 [0.5]	2.177 (1.552;3.068) dominant	2.37 (0.6427)	G^3^	0.878 (1)
6	*HLA-DQA2*	rs9276431	**C**/T	35.5/45.9	203 [41.9]/121 [27.4]	218 [45.0]/236 [53.4]	63 [13.0]/85 [19.2]	0.522 (0.392;0.694) dominant	3.45 (0.7697)	T^3^	0.967 (1)
4	*none*	rs10008032	**C/**T	44.9/53.2	157 [32.4]/84 [19.0]	221 [45.6]/246 [55.7]	107 [22.1]/112 [25.3]	0.490 (0.357;0.671) dominant	3.58 (0.7825)	T	0.591 (1)

1 genes or transcripts according to the UCSC Genome Bioinformatics (http://genome.ucsc.edu/): ***FTO***: fat mass and obesity associated; ***C20orf75***: hypothetical protein LOC164312; ***TSHR***: thyroid stimulating hormone receptor isoform 1; ***BC041448***: Homo sapiens cDNA clone IMAGE:5170949, partial cds; ***PCSK2***: Homo sapiens cDNA FLJ34186 fis; ***HLA-DQA2***: major histocompatibility complex, class II, DQ; none: no gene 250 kb up- or downstream of the SNP,

2 FBATs were all evaluated under additive genetic model (as it is e.g. unknown if a dominant model is also appropriate for the analysis of the family-based data),

3 SNPs showed evidence for a deviation from HWE in founders (p ≤ 0.05),

4 not genotyped due to strong linkage disequilibrium to other *FTO* SNPs,

5 rs41492957according to NCBI Build 36.2

Eleven of the best 15 markers were subsequently genotyped in 644 independent obesity families based on at least one young obese index patient; of the six positive *FTO* SNPs belonging to the same LD block, we genotyped only the two SNPs with the lowest p-values. Confirmation of the initial finding using FBAT (family-based association test) was detected for the two *FTO* SNPs (both Bonferroni corrected p≤0.01). However, none of the risk alleles of the nine other SNPs showed evidence for association in the independent families.

Additionally, similar to the approach of Scuteri et al. [8], we analysed specific candidate genes (coding sequence plus approximately 50 kb flanking the 5′ and 3′ regions, respectively). We chose those candidate genes delineated in the current version of the *Obesity Gene Map Database* (http://obesitygene.pbrc.edu/; [9]), for which two or more independent positive associations to obesity have been reported in addition to those genes listed in the Database to harbour mutations leading to monogenic forms of obesity. In Table S1 (Supporting Information), for each of these genes, the SNP with the lowest p-value and the respective genetic model are shown. Overall, among the 745 SNPs tested 75 (12) had a p-value below 0.05 (0.01). We re-assessed the original publications for the markers in Table S1 (Supporting Information) which had a nominal p-value below 0.005. For three SNPs of the previous publications (*ESR1*: rs2234693; rs9340799 and *LDLR* rs688) HapMap data were available. Hence, we checked for linkage disequilibrium by D' and r^2^. For *ESR1* both previously investigated SNPs (rs2234693; rs9340799 [10], [11]) were approximately 16 kb apart from the 500k SNP rs712221. D' was 0.321 and 0.809, respectively, r^2^ was 0.103 and 0.378, respectively. For *LDLR* the previous SNP (rs688 [12], [13]) was just 48 bp apart from the 500k SNP rs1799898. D' was 1 and r^2^ was 0.144. However, the allele frequencies of the original SNPs and the closest GWA SNPs were quite different (e.g. rs1799898 MAF approximately 12% versus a MAF of 45% in rs688), complicating statements dealing with whether or not both markers tag the same disease related haplotype. Clearly, this possibility requires further attention.

## Discussion

Here we show by a GWA including early onset extremely obese cases (mean BMI Zscore 4.63±2.27) and healthy underweight controls (mean BMI Zscore −1.38±0.35; BMI<15^th^ age percentile) that variation in *FTO* strongly contributes to the development of early onset obesity. Recently, the *FTO* gene was found to be associated with T2DM as based on two GWAs [5], [6]. However, after adjusting for BMI the T2DM association vanished indicating that *FTO* explains variation of body weight. Confirmation in 13 samples with 38,759 individuals and a meta-analysis showed that the A-allele of the variant rs9939609 is associated with a 31% increased risk to develop obesity [5]. These results were independently supported in 8,000 individuals from different populations [14] and in a GWA for obesity-related traits in an epidemiological cohort [8]. The best SNP rs1421085 of the study of Dina et al. [14] showed a nominal p = 3.46×10^−7^ (log-additive OR for the risk allele 1.69, 95% CI 1.38–2.06) in a case-control sample which also comprised obese German children. For our best SNP rs1121980, which is located 8.3 kb upstream of rs1421085 (pairwise r^2^ = 0.90 in CEU HAPMAP; both within intron 1), we found similar estimated genetic effect sizes. As effect sizes for the best markers derived from GWA data sets are usually overestimated [15], our GWA data is an example that this will not always be the case.

Given the relatively small sample size used in our GWA, this investigation nevertheless revealed a single SNP in *FTO* that remained significant after a proper control of the type I error. The *FTO* SNPs have previously been shown to be relevant for obesity in both children and adults [5], [8], [14]. To determine, if the finding is present in all children or only among the older teenagers we did a median split for age within the case group and explored the relationship of each subgroup in comparison to controls as well as to each other (data not shown). The effect is valid in both subgroups and there is no difference between the subgroups. Frayling et al. reported that the association is relevant by the age of 7 and persists into the pre-pubertal period and beyond [5]. Only a meta-analysis addressing developmental aspects will be able to pinpoint, if the effect of the *FTO* variants is more relevant for children or for adults.

Confirmation of the 11 SNPs genotyped in 644 independent obesity families succeeded only for the two *FTO* SNPs (Table 1). Hence, our data suggest that of the best 15 SNPs of the GWA only the *FTO* SNPs represent true positive findings. This is in accordance with a population-based GWA for body weight that also merely resulted in the initial confirmation of only one candidate gene [8].

Our data pertaining to the candidate gene analyses (Table S1) are not readily comparable with the previous publications, as for instance the number of analysed individuals was quite low for some of the previous reports. We restricted our analyses to genes listed in the *Obesity Gene Map Database* (http://obesitygene.pbrc.edu/; [9]) with at least two confirmations; the quality of the original reports varied considerably and for some of the genes different SNPs/variants had been analysed. Hence, we suggest that the candidate genes with SNPs resulting in nominal p-values below 0.005 in our scan should be followed up in subsequent studies.

In general, this report is another proof of concept in favour of GWAs contributing to the investigation of common variation in complex phenotypes.

## Methods

### Participants

487 extremely obese children and adolescents (‘cases’) were recruited in hospitals specialized for the inpatient treatment of extreme obesity (Table 2; mean BMI Z score: 4.63±2.27) while 442 healthy lean individuals (‘controls’) were ascertained at the University of Marburg (Table 2). We relied on older healthy underweight controls to substantially reduce the probability of their becoming overweight and to increase power [e.g. 16]. Based on self-reported questionnaire data on body-weight course, 78% of the lean controls reported having had a below average body weight at age 15, which is similar to the mean age of our obese cases. Thus, our control group mainly comprises individuals who presumably also were in the lower body weight range during adolescence. Details on power considerations are provided in the Supporting Information (Text S1). Written informed consent was given by all participants and in case of minors their parents. The study was approved by the Ethics Committees of the Universities of Marburg and Essen and conducted in accordance with the guidelines of *The Declaration of Helsinki*.

**Table 2:** Description of samples genotyped with the Genome-Wide Human SNP Array 5.0 and the obesity families used for confirmation of the 11 best SNPs

population		status	n total (female)	age [years] (mean; SD)	BMI [m/kg^2^] (mean; SD)	BMI Zscore (mean; SD)	Waist-hip ratio (mean; SD)
*GWA*							
	German extremely obese children and adolescents	cases	487 (278)	14.38 (3.74)	33.40 (6.81)	4.63 (2.27)	0.90 (0.11)
	German lean subjects	controls	442 (271)	26.07 (5.79)	18.31 (1.10)	−1.38 (0.35)	0.77 (0.06)
*confirmation family-based association study*							
	German extremely obese children and adolescents	index patients	644 (363)	13.55 (2.91)	31.92 (5.96)	4.20 (2.01)	0.90 (0.09)
	German obese children and adolescents	siblings	337 (181)	14.91 (5.09)	27.86 (5.24)	2.80 (1.62)	0.88 (0.09)
	parents of the obese children and adolescents	parents	1288 (644)	42.99 (5.92)	30.44 (6.26)	1.69 (1.83)	0.91 (0.11)

### Genotyping

Genotyping was performed on the Genome-Wide Human SNP Array 5.0 (http://www.affymetrix.com/) at the Affymetrix Services Lab (California, USA). 440,794 genotypes of 929 individuals (Dynamic Model algorithm call rate>86%) were called by the BRLMM-P algorithm. For the replication of 11 SNPs genotyping was performed by matrix-assisted laser desorption ionization-time of flight mass spectrometry (MALDI-TOF MS) analysis of allele-dependent primer extension products as described elsewhere [17].

### Statistical Methods

For the GWA data, SNPs with a call rate<95%, departure from Hardy-Weinberg equilibrium in the control group (exact test p<0.01), or with minor allele frequency below 10 percent were excluded from the final analysis (151,503 excluded; 289,291 retained; see Supporting Information; Text S1 and Table S2). The statistical analyses followed the procedure of Sladek et al. [4]. Details are described in the Supporting Information (Text S1). All reported nominal p-values of the GWA are two-sided and asymptotic. In addition, empirical p-values corrected for genome-wide testing and maximization across genetic models are provided. We used a genome-wide significance level of .05 (two-sided). For the confirmation study, both nominal one-sided and Bonferroni corrected (11 tests) p-values are presented for the risk alleles identified in the GWA.

### Confirmation

The 11 of the 15 best SNPs (ranked by p-value, irrespective of the genetic model; see Table 1) were genotyped in 644 independent obesity families comprising 644 extremely obese children and adolescents (index patients) and both of their biological parents; additionally in 297 families obese sibs were also included (for details see Table 2). As none of the 11 SNPs showed strong evidence for a recessive genetic model in the GWA, it was decided to restrict the family-based association testing to the additive model for each of the SNPs (FBAT additive) in order to reduce the amount of multiple testing.

### Candidate gene analyses

Within the GWA data we analysed genes previously suggested to be involved in body weight regulation. We examined 745 SNPs (located within the gene and approximately 50kb 5′and 50kb 3′to the gene) in 47 candidate genes (single gene mutations with an obesity phenotype and candidate genes associated with obesity in at least two independent studies as shown in the *Obesity Gene Map Database*; http://obesitygene.pbrc.edu/) and determined the number of SNPs with p-values ≤ 0.05 (0.01). In addition, we provided information on the SNP with the lowest p-value among all tested genetic models for the respective candidate gene in Table S1 (Supporting Information). For markers which had a nominal p-value below 0.005 we re-assessed the original publications in order to figure out if the marker in our GWA scan matches the information provided in the original publications.

## Supporting Information

Table S1 Analyses of obesity candidate genes (according to the *human obesity gene map*: the 2005 update: Rankinen et al., 2006) in the GWA approach
(0.16 MB DOC)

Table S2 Genotyping and quality control
(0.08 MB DOC)

Text S1 Genotyping and quality control; Additional information on statistical analyses; References for the Supporting Information.
(0.04 MB DOC)
