# Supplementary material for: Genome Wide Association (GWA) Study for Early Onset Extreme Obesity Supports the Role of Fat Mass and Obesity Associated Gene (FTO) Variants
Source: PLoS One. 2007 Dec 26;2(12):e1361. doi: 10.1371/journal.pone.0001361 (PMC2137937; doi:10.1371/journal.pone.0001361)
Supplement: Table S1 — Analyses of obesity candidate genes (according to the human obesity gene map: the 2005 update: Rankinen et al., 2006) in the GWA approach (0.16 MB DOC) [file pone.0001361.s001.doc]

**Table S1 Analyses of obesity candidate genes (according to the *human obesity gene map: the 2005 update*: Rankinen et al., 2006)** in the GWA approach

| chromosome | Gene according to *the human obesity gene map (2005 update)** | SNP | GWA 500K case-control approach | | | | | |
| --- | --- | --- | --- | --- | --- | --- | --- | --- |
|  |  |  | alleles | minor allele frequency in cases  /  controls [%] | genotype distribution cases[%]  /  controls[%] | | | minimal nominal p-value |
| [chr17:58915909-58952935](http://genome.ucsc.edu/cgi-bin/hgTracks?position=chr17:58915909-58952935&hgsid=97824348&knownGene=pack&hgFind.matches=uc002jaw.1,) | ***ACE*** | rs4351 | A/G | 45.0/47.7 | 133  [27.4]  /  126  [28.5] | 268  [55.1]  /  210  [47.5] | 85  [17.5]  /  106  [24.0] | 0.0145 (dom) |
| chr2:254872-268282 | ***ACP1*** | rs10188763 | A/G | 34.7/36.8 | 207  [42.5]  /  176  [39.8] | 222  [45.6]  /  206  [46.6] | 58  [11.9]  /  60  [13.6] | 0.3327 (add) |
| [chr3:188043157-188058946](http://genome.ucsc.edu/cgi-bin/hgTracks?position=chr3:188043157-188058946&hgsid=97824387&knownGene=pack&hgFind.matches=uc003fra.1,) | ***ADIPOQ*** | rs3774261 | A/G | 41.0/36.1 | 158  [32.6]  /  179  [40.6] | 255  [52.7]  /  205  [46.5] | 71  [14.7]  /  57  [12.9] | 0.0121 (dom) |
| chr5:148186349-148188381 | ***ADRB2*** | rs2400707 | A/G | 36.8/42.4 | 194  [39.8]  /  140  [32.0] | 228  [46.8]  /  225  [51.4] | 65  [13.3]  /  73  [16.7] | 0.0127 (log-additive) |
| chr8:37939673-37943341 | ***ADRB3*** | rs4956542 | C/T | 13.9/14.5 | 364  [75.1]  /  323  [73.1] | 107  [22.1]  /  109  [24.7] | 14  [2.9]  /  10  [2.3] | 0.4930 (dom) |
| [chr16:66073975-66075217](http://genome.ucsc.edu/cgi-bin/hgTracks?position=chr16:66073975-66075217&hgsid=97824387&knownGene=pack&hgFind.matches=uc002etg.1,) | ***AGRP*** | no SNP with MAF >10% |  |  |  |  |  |  |
| [chr11:116196628-116199221](http://genome.ucsc.edu/cgi-bin/hgTracks?position=chr11:116196628-116199221&hgsid=97824387&knownGene=pack&hgFind.matches=uc001pps.1,) | ***APOA4*** | rs7396851 | C/T | 16.5/15.4 | 338  [69.7]  /  313  [70.8] | 133  [27.4]  /  121  [27.4] | 14  [2.9]  /  8  [1.8] | 0.2820 (rec) |

- will be continued –

- Table S1 continued -

| [chr2:21077806-21120450](http://genome.ucsc.edu/cgi-bin/hgTracks?position=chr2:21077806-21120450&hgsid=97824387&knownGene=pack&hgFind.matches=uc002red.1,) | ***APOB*** | rs6544366 | G/T | 27.7/24.7 | 287  [58.9]  /  253  [57.2] | 178  [36.6]  /  159  [36.0] | 22  [4.5]  /  30  [6.8] | 0.1328 (dom) |
| --- | --- | --- | --- | --- | --- | --- | --- | --- |
| [chr17:41217449-41268973](http://genome.ucsc.edu/cgi-bin/hgTracks?position=chr17:41217449-41268973&hgsid=97824387&knownGene=pack&hgFind.matches=uc002ijm.1,) | [***CRHR1***](http://obesitygene.pbrc.edu/cgi-bin/ace/gene/acedb?name=CRHR1) | rs17763104 | A/G | 13.0/14.5 | 364  [74.7]  /  324  [73.3] | 119  [24.4]  /  107  [24.2] | 4  [0.8]  /  11  [2.5] | 0.0440 (dom) |
| [chr7:30658725-30706244](http://genome.ucsc.edu/cgi-bin/hgTracks?position=chr7:30658725-30706244&hgsid=97824387&knownGene=pack&hgFind.matches=uc003tbp.1,) | [***CRHR2***](http://obesitygene.pbrc.edu/cgi-bin/ace/gene/acedb?name=CRHR2) | rs975537 | A/T | 25.0/25.4 | 265  [55.1]  /  241  [56.4] | 191  [39.7]  /  155  [36.3] | 25  [5.2]  /  31  [7.3] | 0.1972 (rec) |
| chr8:143988977-143996261 | ***CYP11B2*** | rs3753122 | G/T | 49.7/47.1 | 125  [25.8]  /  132  [29.9] | 236  [48.8]  /  203  [45.9] | 123  [25.4]  /  107  [24.2] | 0.1705 (dom) |
| [chr6:152170379-152466099](http://genome.ucsc.edu/cgi-bin/hgTracks?position=chr6:152170379-152466099&hgsid=97824387&knownGene=pack&hgFind.matches=uc003qoo.1,) | ***ESR1*** | rs712221 | A/T | 43.0/.36.3 | 158  [32.4]  /  188  [42.5] | 239  [49.1]  /  187  [42.3] | 90  [18.5]  /  67  [15.2] | 0.0014 (dom) |
| [chr4:120457853-120462766](http://genome.ucsc.edu/cgi-bin/hgTracks?position=chr4:120457853-120462766&hgsid=97824387&knownGene=pack&hgFind.matches=uc003icw.1,) | ***FABP2*** | rs17009129 | C/T | 30.9/32.5 | 223  [45.9]  /  210  [47.5] | 225  [46.3]  /  176  [39.8] | 38  [7.8]  /  56  [12.7] | 0.0144 (rec) |
| chr16:85158443-85159948 | ***FOXC2*** | rs3829536 | C/T | 29.2/30.8 | 230  [47.9]  /  220  [50.3] | 219  [45.6]  /  164  [37.5] | 31  [6.5]  /  53  [12.1] | 0.0029 (rec) |
| chr12:6819636-6826818 | ***GNB3*** | rs1075836 | C/G | 32.1/34.0 | 224  [46.2]  /  189  [42.8] | 210  [43.3]  /  205  [46.4] | 51  [10.5]  /  48  [10.9] | 0.2946 (dom) |
| chr22:39405128-39408764 | [***GPR24***](http://obesitygene.pbrc.edu/cgi-bin/ace/gene/acedb?name=GPR24) | rs133074 | C/T | 47.8/44.2 | 137  [28.5]  /  137  [31.9] | 228  [47.4]  /  204  [47.6] | 116  [24.1]  /  88  [20.5] | 0.1317 (allelic) |
| chr7:22733323-22738141 | ***IL6*** | rs1476483 | A/G | 18.8/19.3 | 312  [64.1]  /  289  [65.4] | 166  [34.1]  /  135  [30.5] | 9  [1.8]  /  18  [4.1] | 0.0438 (dom) |

- will be continued –

- Table S1 continued -

| chr1:152644293-152706812 | ***IL6R*** | rs6696089 | A/G | 25.1/28.7 | 273  [56.3]  /  214  [48.5] | 180  [37.1]  /  200  [45.4] | 32  [6.6]  /  27  [6.1] | 0.0181 (dom) |
| --- | --- | --- | --- | --- | --- | --- | --- | --- |
| chr11:2137585-2139015 | ***INS*** | rs6578985 | A/G | 30.0/31.5 | 237  [48.7]  /  213  [48.2] | 207  [42.5]  /  179  [40.5] | 43  [8.8]  /  50  [11.3] | 0.2080 (dom) |
| chr2:227308182-227372719 | ***IRS1*** | rs1922531 | G/T | 41.8/37.3 | 162  [33.4]  /  181  [41.0] | 240  [49.5]  /  192  [43.4] | 83  [17.1]  /  69  [15.6] | 0.0174 (dom) |
| chr19:11061132-11105490 | ***LDLR*** | rs1799898 | C/T | 12.5/12.4 | 380  [78.0]  /  334  [75.6] | 92  [18.9]  /  106  [24.0] | 15  [3.1]  /  2  [0.5] | 0.0028 (rec) |
| chr7:127668567-127684917 | [***LEP***](http://obesitygene.pbrc.edu/cgi-bin/ace/gene/acedb?name=LEP) | rs12706832 | C/T | 44.7/47.0 | 154  [32.0]  /  113  [25.8] | 225  [46.7]  /  238  [54.3] | 103  [21.4]  /  87  [19.9] | 0.0400 (dom) |
| chr1:65658906-65875410 | [***LEPR***](http://obesitygene.pbrc.edu/cgi-bin/ace/gene/acedb?name=LEPR) | rs2025805 | C/T | 40.6/46.5 | 169  [35.9]  /  125  [29.6] | 221  [46.9]  /  202  [47.8] | 81  [17.2]  /  96  [22.7] | 0.0117 (allelic) |
| [chr15:56511467-56648364](http://genome.ucsc.edu/cgi-bin/hgTracks?position=chr15:56511467-56648364&hgsid=97824387&knownGene=pack&hgFind.matches=uc002afa.1,) | ***LIPC*** | rs9920144 | C/T | 33.0/ 28.6 | 211  [43.5]  /  224  [50.7] | 222  [45.8]  /  183  [41.4] | 52  [10.7]  /  35  [7.9] | 0.0195 (add) |
| chr19:47597506-47623418 | ***LIPE*** | rs8112156 | C/T | 11.2/10.6 | 388  [79.7]  /  353  [79.9] | 88  [18.1]  /  84  [19.0] | 11  [2.3]  /  5  [1.1] | 0.1871 (rec) |
| chr1:154318993-154376495 | ***LMNA*** | rs12063564 | C/T | 4.3/13.5 | 359  [73.7]  /  328  [74.2] | 116  [23.8]  /  108  [24.4] | 12  [2.5]  /  6  [1.4] | 0.2216 (rec) |
| [chr8:19841058-19869049](http://genome.ucsc.edu/cgi-bin/hgTracks?position=chr8:19841058-19869049&hgsid=97813142&knownGene=pack&hgFind.matches=uc003wzk.1,) | ***LPL*** | rs11986942 | C/G | 31.7/29.0 | 241  [49.7]  /  224  [50.7] | 180  [37.1]  /  179  [40.5] | 64  [13.2]  /  39  [8.8] | 0.0343 (rec) |

- will be continued –

- Table S1 continued -

| chr20:54257196-54258278 | ***MC3R*** | rs1326022 | C/T | 25.8/30.9 | 276  [56.7]  /  207  [46.8] | 170  [34.9]  /  196  [44.3] | 41  [8.4]  /  39  [8.8] | 0.0027 (dom) |
| --- | --- | --- | --- | --- | --- | --- | --- | --- |
| chr18:56189544-56190981 | ***MC4R*** | rs17066829 | A/T | 36.2/32.5 | 199  [41.2]  /  203  [46.1] | 218  [45.1]  /  188  [42.7] | 66  [13.7]  /  49  [11.1] | 0.0919 (allelic) |
| chr7:24290334-24298002 | ***NPY*** | rs6461787 | A/C | 40.9/35.4 | 177  [36.4]  /  190  [43.0] | 220  [45.3]  /  191  [43.2] | 89  [18.3]  /  61  [13.8] | 0.0142 (allelic) |
| [chr1:27110566-27113047](http://genome.ucsc.edu/cgi-bin/hgTracks?position=chr1:27110566-27113047&hgsid=97824387&knownGene=pack&hgFind.matches=uc001bnf.1,) | ***NR0B2*** | rs17162316 | C/G | 15.5/18.3 | 344  [71.4]  /  290  [66.2] | 126  [26.1]  /  135  [30.8] | 12  [2.5]  /  13  [3.0] | 0.0913 (dom) |
| [chr5:142637689-142795270](http://genome.ucsc.edu/cgi-bin/hgTracks?position=chr5:142637689-142795270&hgsid=97824387&knownGene=pack&hgFind.matches=uc003lne.1,) | ***NR3C1*** | rs2963154 | C/T | 13.8/14.9 | 358  [73.5]  /  323  [73.1] | 123  [25.3]  /  106  [24.0] | 6  [1.2]  /  13  [2.9] | 0.0660 (dom) |
| chr9: 86473286-86828325 | ***NTRK2*** | rs10780688 | A/G | 45.9/51.1 | 133  [27.5]  /  105  [23.9] | 256  [53.0]  /  219  [49.9] | 94  [19.5]  /  115  [26.2] | 0.0147 (rec) |
| chr5:95751875-95794708 | ***PCSK1*** | rs266695 | C/T | 32.5/27.8 | 221  [45.6]  /  230  [52.3] | 212  [43.7]  /  175  [39.8] | 52  [10.7]  /  35  [8.0] | 0.0268 (allelic) |
| chr15:88008603-88023595 | ***PLIN*** | rs2119606 | A/G | 31.4/35.9 | 229  [47.4]  /  188  [42.8] | 204  [42.2]  /  186  [42.4] | 50  [10.4]  /  65  [14.8] | 0.0401 (allelic) |
| [chr2:25237226-25245063](http://genome.ucsc.edu/cgi-bin/hgTracks?position=chr2:25237226-25245063&hgsid=97824387&knownGene=pack&hgFind.matches=uc002rga.1,) | ***POMC*** | rs7565877 | A/G | 9.1/10.9 | 400  [82.1]  /  348  [78.7] | 85  [17.5]  /  91  [20.6] | 2  [0.4]  /  3  [0.7] | 0.1765 (add) |
| [chr22:44925163-45018317](http://genome.ucsc.edu/cgi-bin/hgTracks?position=chr22:44925163-45018317&hgsid=97824387&knownGene=pack&hgFind.matches=uc003bgw.1,) | ***PPARA*** | rs5768939 | A/G | 15.4/15.0 | 342  [70.4]  /  321  [72.6] | 138  [28.4]  /  109  [24.7] | 6  [1.2]  /  12  [2.7] | 0.1024 (rec) |

- will be continued –

- Table S1 continued -

| chr6:35418313-35503933 | ***PPARD*** | rs7758978 | C/G | 40.2/37.2 | 183  [37.6]  /  168  [38.0] | 216  [44.4]  /  219  [49.5] | 88  [18.1]  /  55  [12.4] | 0.0176 (dom) |
| --- | --- | --- | --- | --- | --- | --- | --- | --- |
| [chr3:12304349-12450855](http://genome.ucsc.edu/cgi-bin/hgTracks?position=chr3:12304349-12450855&hgsid=97824387&knownGene=pack&hgFind.matches=uc003bwr.1,) | ***PPARG*** | rs1801282 | C/G | 14.1/11.1 | 354  [72.8]  /  345  [78.1] | 126  [25.9]  /  95  [21.5] | 6  [1.2]  /  2  [0.5] | 0.0451 (add) |
| [chr4:23402742-23500798](http://genome.ucsc.edu/cgi-bin/hgTracks?position=chr4:23402742-23500798&hgsid=97813918&knownGene=pack&hgFind.matches=uc003gqs.1,) | ***PPARGC1A*** | rs4697417 | A/G | 42.4/45.5 | 160  [33.4]  /  117  [27.0] | 231  [48.2]  /  239  [55.1] | 88  [18.4]  /  78  [18.0] | 0.0344 (dom) |
| [chr20:48560298-48634493](http://genome.ucsc.edu/cgi-bin/hgTracks?position=chr20:48560298-48634493&hgsid=97824387&knownGene=pack&hgFind.matches=uc002xvl.1,) | ***PTPN1*** | rs12479940 | A/T | 31.6/34.2 | 229  [47.0]  /  189  [42.8] | 208  [42.7]  /  203  [45.9] | 50  [10.3]  /  50  [11.3] | 0.1921 (dom) |
| [chr19:7639972-7641340](http://genome.ucsc.edu/cgi-bin/hgTracks?position=chr19:7639972-7641340&hgsid=97824387&knownGene=pack&hgFind.matches=uc002mhg.1,) | ***RETN*** | rs10518270 | A/G | 14.5/12.6 | 258  [66.8]  /  334  [75.6] | 115  [29.8]  /  104  [23.5] | 13  [3.4]  /  4  [0.9] | 0.0446 (rec) |
| chr6:100943471-101018272 | ***SIM1*** | rs9386144 | C/T | 19.3/ 23.6 | 312  [64.1]  /  261  [59.1] | 162  [33.3]  /  153  [34.6] | 13  [2.7]  /  28  [6.3] | 0.0065 (rec) |
| chr6:31651329-31654091 | ***TNF*** | rs1046089 | A/G | 34.6/30.4 | 201  [41.6]  /  203  [46.3] | 229  [47.4]  /  203  [46.3] | 53  [11.0]  /  32  [7.4] | 0.0470 (add) |
| [chr11:73363364-73371537](http://genome.ucsc.edu/cgi-bin/hgTracks?position=chr11:73363364-73371537&hgsid=97824387&knownGene=pack&hgFind.matches=uc001ouq.1,) | ***UCP2*** | rs1800849 | C/T | 20.3/25.6 | 310  [63.7]  /  242  [54.8] | 156  [32.0]  /  173  [39.1] | 21  [4.3]  /  27  [6.1] | 0.0057 (dom) |
| chr11:73388985-73397778 | ***UCP3*** | rs1800849 | C/T | 20.3/25.6 | 310  [63.7]  /  242  [54.8] | 156  [32.0]  /  173  [39.1] | 21  [4.3]  /  27  [6.1] | 0.0057 (dom) |
| [chr12:46521587-46585081](http://genome.ucsc.edu/cgi-bin/hgTracks?position=chr12:46521587-46585081&hgsid=97824387&knownGene=pack&hgFind.matches=uc001rqm.1,) | ***VDR*** | rs7418 | C/T | 25.8/21.3 | 268  [55.0]  /  273  [61.8] | 186  [38.2]  /  149  [33.7] | 33  [6.8]  /  20  [4.5] | 0.0230 (allelic) |

- will be continued -

- Table S1 continued –

The reported minimal p-values are nominal and reported independently of the underlying genetic model. *Best minimal nominal p-values of candidate genes for obesity (Rankinen et al., 2006: The human obesity gene map: the 2005 update). Single gene mutations with an obesity phenotype and candidate genes with at least two positive associations with BMI were included. We selected all SNPs located in the chromosomal region of the gene (according to Golden Path ([http://genome.ucsc.edu](http://genome.ucsc.edu/))) and about 50kb 5´and 3´of the gene. All alleles were transcribed to the forward strand.

Gene abbreviations are as follows: ***ACE****: angiotensin I-converting enzyme;* ***ACP1:*** *acid phosphatase 1, soluble;* ***ADIPOQ:*** *adipocyte, C1Q, and collagen domain containing;* ***ADRB2:*** *beta-2-adrenergic receptor;* ***ADRB3:*** *beta-3-adrenergic receptor;* ***AGRP:*** *agouti-related protein, mouse, homolog of;* ***APOA4:*** *apolipoprotein A-IV;* ***APOB:*** *apolipoprotein B;* [***CRHR1***](http://obesitygene.pbrc.edu/cgi-bin/ace/gene/acedb?name=CRHR1)***:*** *corticotropin-releasing hormone receptor 1;* [***CRHR2***](http://obesitygene.pbrc.edu/cgi-bin/ace/gene/acedb?name=CRHR1)***:*** *corticotropin-releasing hormone receptor 2;* ***CYP11B2:*** *cytochrome P450, subfamily xib, polypeptide 2;* ***ESR1:*** *estrogen receptor 1;* ***FABP2:*** *fatty acid-binding protein 2;* ***FOXC2:*** *forkhead box C2;* ***GNB3:*** *guanine nucleotide-binding protein, beta-3;* ***GPR24****: G-protein-coupled receptor 24;* ***IL6:*** *interleukin 6;* ***IL6R:*** *interleukin 6 receptor;* ***INS****: insulin;* ***IRS1:*** *insulin receptor substrate 1;* ***LDLR:*** *low density lipoprotein receptor;* [***LEP***](http://obesitygene.pbrc.edu/cgi-bin/ace/gene/acedb?name=LEP)*: leptin;* [***LEPR***](http://obesitygene.pbrc.edu/cgi-bin/ace/gene/acedb?name=LEPR)***:*** *leptin receptor;* ***LIPC:*** *lipase, hepatic;* ***LIPE****: lipase, hormone-sensitive;* ***LMNA:*** *lamin A/C;* ***LPL:*** *lipoprotein lipase;* ***MC3R:*** *melanocortin 3 receptor;* ***MC4R****: melanocortin 4 receptor;* ***NPY:*** *neuropeptide Y;* ***NR0B2:*** *nuclear receptor subfamily 0, group B, member 2;* ***NR3C1:*** *nuclear receptor subfamily 3, group C, member 1/ glucocorticoid receptor;* ***NTRK2:*** *neurotrophic tyrosine kinase, receptor, type 2;* ***PCSK1:*** *proprotein convertase, subtilisin/kexin-type, 1;* ***PLIN:*** *perilipin;* ***POMC:*** *proopiomelanocortin;* ***PPARA:*** *peroxisome proliferator-activated receptor-alpha;* ***PPARD:*** *peroxisome proliferator-activated receptor-delta;* ***PPARG:*** *peroxisome proliferator-activated receptor-gamma;* ***PPARGC1A:*** *peroxisome proliferator-activated receptor-gamma, coactivator 1, alpha;* ***PTPN1:*** *protein-tyrosine phosphatase, nonreceptor-type, 1;* ***RETN:*** *resistin;* ***SIM1:*** *single-minded, drosophila, homolog of, 1;* ***TNF:*** *tumor necrosis factor;* ***UCP2:*** *uncoupling protein 2;* ***UCP3:*** *uncoupling protein 3;* ***VDR:*** *vitamin D receptor*
