# Supplementary material for: Genome Wide Association (GWA) Study for Early Onset Extreme Obesity Supports the Role of Fat Mass and Obesity Associated Gene (FTO) Variants
Source: PLoS One. 2007 Dec 26;2(12):e1361. doi: 10.1371/journal.pone.0001361 (PMC2137937; doi:10.1371/journal.pone.0001361)
Supplement: Table S2 — Genotyping and quality control (0.08 MB DOC) [file pone.0001361.s002.doc]

**Table S2 Genotyping and quality control**

| chromosome | number of SNPs | number of SNPs excluded | percent excluded | percent excluded due to filter* | | |
| --- | --- | --- | --- | --- | --- | --- |
|  |  |  |  | call-rate | HWE | MAF |
| no locus information | 60 | 28 | 46.67 | 3.57 | 32.14 | 85.71 |
| 1 | 35,162 | 12,588 | 35.80 | 4.18 | 5.04 | 95.5 |
| 2 | 36,569 | 12,673 | 34.66 | 4.84 | 5.36 | 94.67 |
| 3 | 30,039 | 9,879 | 32.89 | 4.84 | 5.51 | 94.5 |
| 4 | 28,435 | 10,074 | 35.43 | 5.01 | 4.81 | 95.28 |
| 5 | 28,376 | 9,413 | 33.17 | 4.56 | 5.62 | 94.86 |
| 6 | 27,777 | 8,784 | 31.62 | 4.66 | 6.36 | 94.27 |
| 7 | 22,694 | 7,361 | 32.44 | 4.84 | 6.49 | 94.24 |
| 8 | 24,338 | 8,269 | 33.98 | 4.70 | 5.71 | 94.5 |
| 9 | 20,016 | 6,860 | 34.27 | 4.88 | 5.25 | 94.71 |
| 10 | 25,034 | 8,774 | 35.05 | 4.35 | 5.22 | 95.28 |
| 11 | 22,952 | 7,765 | 33.83 | 5.05 | 5.52 | 94.57 |
| 12 | 21,810 | 7,543 | 34.59 | 4.65 | 5.59 | 94.84 |
| 13 | 16,943 | 5,992 | 35.37 | 4.84 | 5.21 | 94.91 |
| 14 | 13,842 | 4,928 | 35.60 | 4.18 | 6.72 | 93.93 |
| 15 | 12,628 | 4,592 | 36.36 | 4.55 | 5.23 | 94.95 |
| 16 | 13,397 | 4,698 | 35.07 | 4.62 | 5.98 | 94.66 |
| 17 | 9819 | 3,280 | 33.40 | 5.03 | 5.61 | 94.48 |
| 18 | 13,136 | 4,711 | 35.86 | 4.08 | 5.86 | 94.63 |
| 19 | 5,440 | 1,776 | 32.65 | 4.79 | 4.84 | 94.99 |
| 20 | 10,976 | 3,822 | 34.82 | 4.26 | 7.01 | 94.09 |
| 21 | 6,235 | 2,056 | 32.98 | 4.57 | 5.35 | 95.23 |
| 22 | 5,330 | 1,895 | 35.55 | 4.80 | 6.54 | 93.72 |
| pseudo-autosomal | 155 | 50 | 32.26 | 6.00 | 10.00 | 90.00 |
| X | 9,631 | 3,692 | 38.33 | 4.25 | 4.98 | 96.24 |
|  |  |  |  |  |  |  |

* among the excluded SNPs, the percentages according to which the SNP was excluded; note that a single SNP may have failed to pass several filters. HWE: Hardy-Weinberg equilibrium; MAF: minor allele frequency.
