# Supplementary material for: Genome Wide Association (GWA) Study for Early Onset Extreme Obesity Supports the Role of Fat Mass and Obesity Associated Gene (FTO) Variants
Source: PLoS One. 2007 Dec 26;2(12):e1361. doi: 10.1371/journal.pone.0001361 (PMC2137937; doi:10.1371/journal.pone.0001361)
Supplement: Text S1 — Genotyping and quality control; Additional information on statistical analyses; References for the Supporting Information. (0.04 MB DOC) [file pone.0001361.s003.doc]

**Genotyping and quality control**

Genotype calling was performed with the BRLMM-P analysis tool [1, 2]. Furthermore, several filters were applied for exclusion of individual single nucleotide polymorphisms (SNPs):

- call-rate (%) < 95 (if the SNP was available in less than 95% of all genotyped individuals)
- exact two-sided test for deviations from Hardy Weinberg equilibrium (HWE; [3]) in the control group < 0.01
- minor allele frequency (MAF) in all individuals < 0.1

A total of 151,503 (34.37%) from the available 440,794 SNPs were excluded from the case-control analysis if one of the filters indicated a violation of the quality. Table S2 provides detailed information for SNPs excluded by chromosome. In addition for all SNPs that came through the above filters and which were among the best SNPs (Table 2) the signal intensity plots were directly visually inspected to exclude possible artefacts due to miscalling.

**Additional information on statistical analyses**

Each of the autosomal/pseudoautosomal SNPs was analyzed using an allelic, a recessive, a log-additive and a dominant genetic mode of inheritance. Similarly all four models were used for analyses of the X-chromosomal SNPs in women whereas for men the analyses were limited to the allelic model. Hence, a total of 1,163,103 tests were considered.

For each SNP, a maximum statistic was determined afterwards as e.g. suggested by [4]. In order to determine empirical p-values for the maximum statistic, case control labels were randomly permuted to generate 10,000 replicates under the global null hypothesis of no genetic association. Within each replicate we again determined the maximum statistic for each SNP. We applied the multiple testing procedure “step-down maxT” described by [5] to determine corrected (empirical) p-values.

Finally, all analysed SNPs were sorted according to the smallest nominal, uncorrected p-value among all results of the genetic models used. In addition empirical p-values corrected for multiple testing across the genome and for multiple genetic models are provided.

It appears in our results that with the permutation method a nominal p-value below 1.13x10^-7^ (best SNP rs1121980) would reach global significance at the 0.05 level, whereas with the Bonferroni correction method a SNP would need to have a nominal p-value below 0.05/1,163,103 =4.30x10^-8^ to be considered as globally significant at the same level.

Power calculations [<http://hydra.usc.edu/gxe>; 6] revealed that the GWA sample has a comparison-wise power ≥ .8 to detect a log-additive OR ≥ 1.5 for minor allele frequencies ≥ .1 (α= .05; two-sided). If maximization across genetic models and multiple testing for all markers is taken into account, a Bonferroni-corrected genome wide significance level of α= 4.30x10^-8^ (two-sided) may be applied. In this case the GWA sample has a comparison-wise power ≥ .8 to detect a log-additive OR ≥ 2 for minor allele frequencies ≥ .2.

**References for the supporting information**

1 Affymetrix White Paper, BRLMM-P: A Genotype-calling Method for the SNP 5.0 Array.

2 Rabbee N, Speed TP (2006) A genotype calling algorithm for affymetrix SNP arrays. Bioinformatics. 22: 7-12.

3 Wigginton JE, Cutler DJ, Abecasis GR (2005) A note on exact tests of Hardy-Weinberg equilibrium. [Am J Hum Genet.](javascript:AL_get(this, 'jour', 'Am J Hum Genet.');) 76: 887-93.

4 Sladek R, Rocheleau G, Rung J, Dina C, Shen L, et al. (2007) A genome-wide association study identifies novel risk loci for type 2 diabetes. Nature. 445: 881-5.

5 Westfall PH, Young SS (1993) Resampling-based multiple testing: examples and methods for p-value adjustment. New York John Wiley & Sons.
